# Clinical and economic burden of anxiety/depression among older adult COPD patients: evidence from the COPD-AD China Registry study

**DOI:** 10.3389/fpsyt.2023.1221767

**Published:** 2024-01-08

**Authors:** Xuanna Zhao, Gege Liu, Dewei Liu, Li Zou, Qiu Huang, Min Chen, Dongming Li, Bin Wu, Hua Wu, Dan Huang, Dong Wu

**Affiliations:** ^1^Department of Respiratory and Critical Care Medicine, Affiliated Hospital of Guangdong Medical University, Zhanjiang, Guangdong, China; ^2^First College for Clinical Medicine, Guangdong Medical University, Zhanjiang, Guangdong, China; ^3^Department of Information Technology, Affiliated Hospital of Guangdong Medical University, Zhanjiang, Guangdong, China

**Keywords:** anxiety, clinical burden, COPD, depression, economic burden

## Abstract

**Background:**

Anxiety and depression are common in patients with chronic obstructive pulmonary disease (COPD), especially older adult patients. This can complicate the disease progression and lead to increased clinical and economic burden. We sought to investigate the clinical and economic burdens associated with the presence of anxious and/or depressive symptoms among older adult COPD patients.

**Methods:**

We screened 579 patients aged over 60 years and diagnosed with COPD via a lung function test following the 2017 Global Initiative Chronic Obstructive Lung Disease (GOLD) guidelines. Anxiety and depression were measured using the Hospital Anxiety and Depression Scale (HADS) through face-to-face interviews at admission. Follow-up was conducted by telephone calls at 6, 12, 18, 24, and 36 months after discharge to assess clinical and economic burden. COPD-anxiety and/or depression patients were matched to patients without anxiety and depression (COPD-only) using propensity scores. Multivariate regression models were used to compare clinical and economic burden between COPD-anxiety and/or depression and COPD-only groups.

**Results:**

Compared with COPD-only patients, COPD patients complicated with anxiety and/or depression had increased clinical burden, including higher COPD-related outpatient visits, COPD-related hospitalizations, and length of COPD-related hospitalizations (*p* < 0.001). Moreover, they also had an increased economic burden, including higher annual total healthcare costs, medical costs, and pharmacy costs (*p* < 0.001).

**Conclusion:**

Older adult COPD patients with anxiety or depression had significantly higher clinical and economic burdens than patients without these comorbidities. These findings deserve further exploration and may be useful for the formulation of relevant healthcare policies.

## 1 Introduction

Chronic obstructive pulmonary disease (COPD) is a chronic disease characterized by persistent airflow restriction. With the rapidly aging population, high prevalence of smoking, and high levels of air pollution, the clinical burden of COPD in China is expected to continue to increase. Individuals with chronic conditions often experience poor mental health (1); older adult COPD patients are more likely to develop mental illnesses, specifically anxiety and depression (2, 3). In a previous study, we also found that 13.93% of patients with stable COPD reported anxious symptoms, and 23.37% experienced depression (4).

For COPD patients, there are many identified risk factors for suffering from anxiety or depression, such as continuous smoking, poor knowledge level, low acceptance of disease, and low socioeconomic status (5–8). More importantly, as COPD progresses, patients experience long-term use of systemic corticosteroids, poorer quality of life, increased breathing difficulties, and severe impairment of physical function, leading to more severe symptoms of anxiety and depression (9–11). The presence of anxiety or depression may impose substantial disease burden, and increases healthcare utilization among older adult COPD patients (12). Either can complicate the course of COPD by causing poor adherence to treatment, as well as increased risks of exacerbation and emergency care use (13–15). Furthermore, they can also affect various aspects of quality of life in older adult COPD patients, such as functional, cognitive, and emotional domains, resulting in more significant functional impairments, more severe disease outcomes, poorer quality of life, and higher risk of death (16–18). All of these conditions may result in additional costs for outpatient visits, emergency room visits, hospitalizations, and prescription expenses, which represent significant clinical and economic burdens for patients, their families, and the whole healthcare system (19–22). Thus, evaluating the burden of mental comorbidities plays an indispensable role in guiding policymakers in allocating resources to achieve optimal treatment goals.

Our previous research has shown that anxiety and depression symptoms are prevalent in COPD patients and are associated with more severe disease outcomes, suggesting that psychological distress may complicate the course of COPD (4). Therefore, we suspect that the combination of anxiety and/or depression symptoms may result in additional visits and medical costs for COPD patients. Although some studies have shown that anxiety and depression increase the clinical and economic burdens of COPD in Europe and America (23, 24), there is a paucity of similar data in China. Therefore, we conducted a study in older adult COPD patients in China to estimate differences in the clinical and economic burdens of patients with or without symptoms of anxiety and/or depression. It is hoped that our study will enable clinicians to pay more attention to anxiety and/or depression in patients with COPD, and to consider the impact of patients’ mental state when formulating relevant healthcare policies and the next version of COPD GOLD guidelines.

## 2 Methods

### 2.1 Data source

The data were sourced from the Registry for Chronic Obstructive Pulmonary Disease With Anxiety and Depression in China (the COPD-AD China Registry) study, a national clinical registration study initiated in June 2017, with a duration of 3.5 years (clinical trial ID: NCT03187236) (25). The study was approved by the institutional review boards of all participating hospitals and carried out in accordance with the Declaration of Helsinki. Written informed consent was obtained from all participating patients before data recording began.

### 2.2 Patient selection

**Figure 1:**
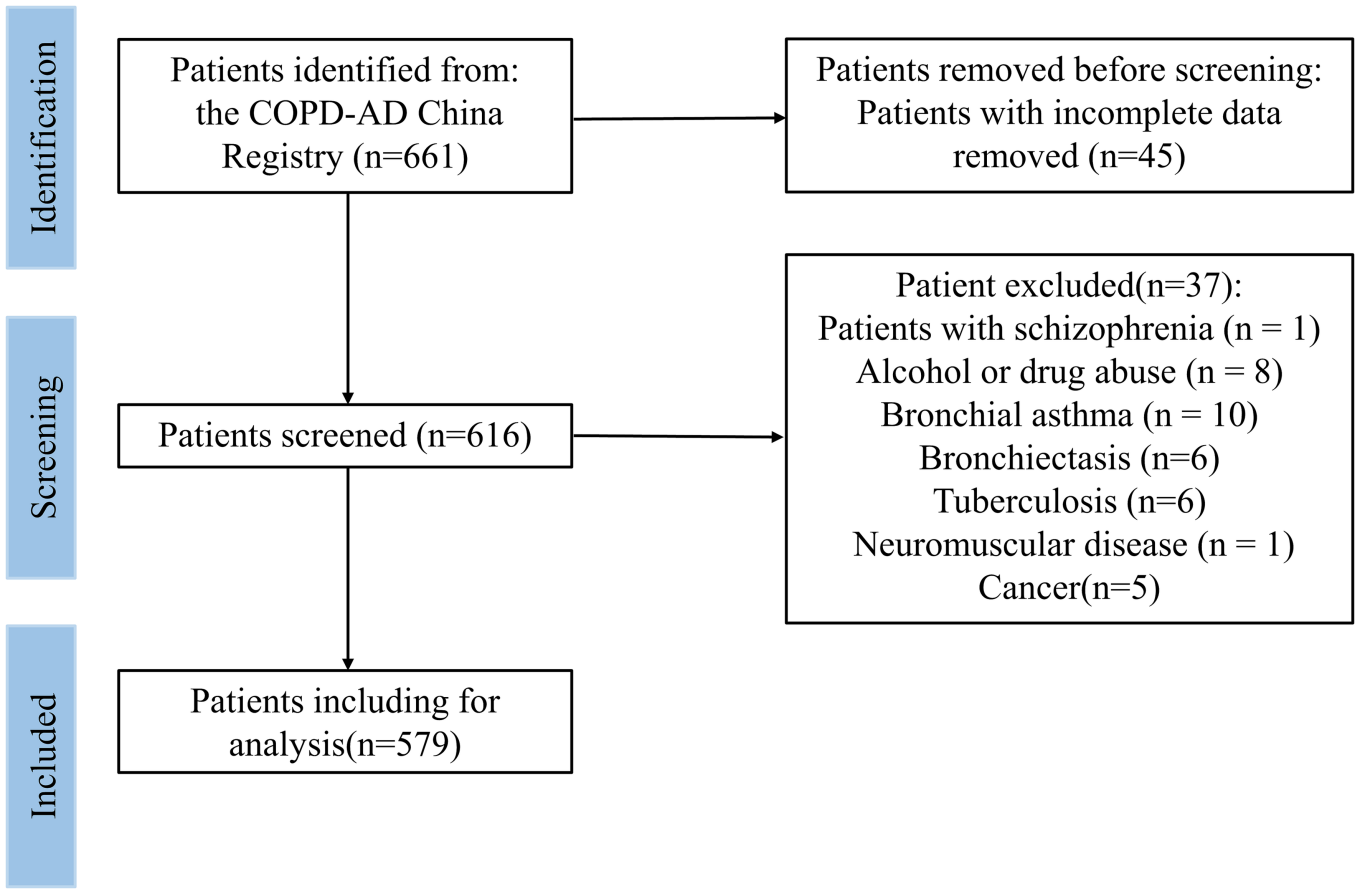
Flow diagram of subjects included in the Patient Registry Study.

In this study, patients from the respiratory department of the Affiliated Hospital of Guangdong Medical University were selected, and their 3-year follow-up information was collected. Face-to-face interviews were conducted at admission, and telephone follow-up was conducted at 6, 12, 18, 24, and 36 months after initial discharge. The interviewer set fixed questions before the interview to ensure that all interviews were similar. Between 2017 and 2021, 661 patients participated in this study. The inclusion criteria were as follows: (1) able to understand the research purpose and sign a consent form; (2) have a diagnosed COPD via a lung function test following the 2017 Global Initiative Chronic Obstructive Lung Disease (GOLD) Global Strategy for the Diagnosis, Management, and Prevention of COPD (26). (3) aged 60 and older. The exclusion criteria were as follows: (1) patients with incomplete data (*n* = 45); (2) pregnancy; (3) severe cognitive disorder; (4) suicide attempt or psychiatric hospitalization in the past year; (5) current suicidal ideation with plan or intent; (6) patients with mental illness, such as dementia, autism, schizophrenia (*n* = 1), or bipolar disorder; (7) patients with other chronic respiratory diseases, such as bronchial asthma (*n* = 10), bronchiectasis (*n* = 6), pulmonary fibrosis, or tuberculosis (*n* = 6); (8) neuromuscular disease (*n* = 1); (9) cancer (*n* = 5). After applying the exclusion criteria, 579 participants were included in the final baseline study sample (Figure 1).

### 2.3 Characteristics and spirometry

Demographic characteristics (age, sex, height, weight, body mass index [BMI], smoking history, education, and household income) and clinical characteristics, including number of acute exacerbations in the past year, COPD Assessment Test (CAT) score, and modified Medical Research Council (mMRC) score, were collected from the data management network.^1^ We performed spirometry according to the guidelines for lung function tests formulated by the Chinese Thoracic Society. We measured the percentage of predicted forced expiratory volume in 1 s (FEV_1_% predicted) for all participating patients (27).

### 2.4 Clinical and economic burden measurements

All COPD-related clinical and economic burdens were calculated after 3 years of follow-up. Specific clinical burden measures included the annual number of COPD-related outpatient visits, COPD-related hospitalizations, and length of COPD-related hospitalizations. Specific economic burden measures included annual COPD-related medical, pharmacy, and total healthcare costs. Costs were presented in Chinese Yuan (CNY). Outpatient visits related to COPD were defined by claims initially diagnosed with COPD. Similarly, hospitalization related to COPD was also defined by claims for initial discharge diagnosis of COPD.

### 2.5 Determination of anxiety and depression

Anxiety and depression were measured using the Hospital Anxiety and Depression Scale (HADS). HADS was developed specifically to detect anxiety and depression in patients with physical disorders (28). It is a proven symptom severity screening tool in cases of anxiety and depression in patients with chronic conditions, including COPD (29, 30). HADS includes 14 items for detecting anxious and depressive symptoms in patients with chronic diseases. There were seven programs each for anxiety and depression, and their total scores are calculated separately. Each item is rated on a Likert scale from 0 to 3. A score of 0–7 indicates no anxiety/depression, while a score greater than 7 indicates anxiety/depression. The higher the score, the greater the likelihood that the patient has anxiety or depression disorders (31).

### 2.6 Propensity score matching

Because this study evaluates the impact of anxiety and depression on clinical and economic burden based on the assumption that all other factors are the same, baseline differences between groups may have the potential to bias the results. Therefore, propensity scores were used to match patients in the COPD-anxiety and/or depression group and the COPD-only group in a 1:1 ratio, with each patient’s score defined as the probability of both anxiety and/or depression under the condition of a baseline variable, including demographic characteristics (i.e., age, sex, BMI, smoking status, number of comorbidities, education, and household monthly income) and clinical characteristics (CAT, mMRC, number of acute exacerbations in the past year, and FEV_1_% predicted). Matching proceeded using the closest available matching technique on the estimated propensity score, performed to 2 decimal places (0.02).

## 3 Statistical analysis

All statistical analyses were performed with IBM SPSS Statistics, Version 25.0 (IBM Corp., Armonk, NY, USA) with the statistical significance level set at *p* < 0.001. Categorical data are reported as numbers (percentages). Continuous variables are presented as means±standard deviation (SD) for normally distributed data or medians (interquartile range [IQR]) for non-normal distributions. The chi-square test was used for comparisons of categorical variables, and the independent *t*-test, Mann–Whitney U test, and Kruskal-Wallis test (ANOVA) were used for the comparison of continuous variables.

After propensity score matching, baseline characteristics were compared between groups using paired *t*-tests or Wilcoxon signed-rank tests (continuous variables) and McNemar’s tests (categorical variables). Adjusted clinical and economic burdens were assessed using multivariate analyses. Covariates with statistical differences were incorporated after matching into the statistical model. Generalized linear models using a gamma distribution with a log link were used to adjust for healthcare costs and medical visits.

## 4 Results

### 4.1 Demographic and clinical characteristics

In total, 579 patients with COPD were included in our analyses; 213 (36.79%) were complicated with anxiety and/or depression. The mean age of the enrolled subjects was 68.75 ± 0.44 years, with men comprising 82.55% of the cohort. We divided all participants into two groups: those without anxiety or depression and those with anxiety and/or depression. At baseline, we observed that the two groups differed significantly in household monthly income, CAT score, and mMRC score among all participants (Table 1).

**Table 1:** Characteristics and symptoms of study participants.

Variables	COPD-only (*n* = 366)	COPD-anxiety and/or depression (*n* = 213)	*p* value
Age, years, mean (SD)	67.98 (0.43)	69.32 (0.59)	0.069
Sex, Male, *n* (%)	306 (83.6)	172 (80.75)	0.051
BMI, kg/m^2^, median (IQR)	19.96 (17.67, 22.31)	19.95 (17.33, 22.40)	0.542
Smoking status, *n* (%)			0.841
Never smoking	70 (19.13)	54 (25.35)	
Current smoking	160 (43.71)	72 (33.80)	
Past smoking	136 (37.16)	87 (40.85)	
Comorbidities, *n* (%)			0.132
<3	356 (97.27)	202 (94.84)	
≥3	10 (2.73)	11 (5.16)	
Education, *n* (%)			0.779
<Senior high school	356 (97.27)	208 (97.65)	
≥Senior high school	10 (2.73)	5 (2.35)	
Household monthly income, *n* (%)			0.009
High	2 (0.55)	1 (0.47)	
Middle	15 (4.10)	21 (9.86)	
Low	349 (95.35)	191 (89.67)	
CAT score, *n* (%)			<0.001
≤20	282 (77.05)	132 (61.97)	
21–40	84 (22.95)	81 (38.03)	
mMRC, *n* (%)			<0.001
0–2	252 (68.85)	109 (51.17)	
3–4	114 (31.15)	104 (48.83)	
Number of acute exacerbations in the past year, *n* (%)			0.065
0–1	275 (75.14)	145 (68.08)	
≥2	91 (24.86)	68 (31.92)	
FEV_1_% predicted, *n* (%)			
≥80	25 (6.83)	23 (10.80)	0.950
≤50 – < 80	127 (34.70)	61 (28.64)	
≤30 – < 50	136 (37.16)	82 (38.50)	
<30	78 (21.31)	47 (22.06)	

Educational status was calculated by having received college education or above. <Senior high school was defined as received secondary education or below; ≥Senior high school was defined as received college education or above. Household monthly income status was calculated from the annual family income using the federal poverty line (FPL). Low was defined as ≤ 100% FPL; middle was defined as > 100% FPL and < 199% FPL; high was defined as ≥ 199% FPL. SD, standard deviation; IQR, interquartile range; BMI, body mass index; CAT, COPD Assessment Test; mMRC, modified Medical Research Council. FEV_1_% predicted, percentage predicted forced expiratory volume in 1 s.

### 4.2 Clinical burden of anxiety and/or depression among COPD patients

The annual number of COPD-related outpatient visits in the COPD-anxiety and/or depression group was 33.33% higher than in the COPD-only group. Additionally, in this group, there were significant increases of 100.00% in the annual number of COPD-related hospitalizations and 50.00% in the length of hospitalizations (Table 2).

**Table 2:** Unadjusted and adjusted clinical burden of anxiety and depression among patients with COPD.

	Unadjusted clinical burden, median (IQR)			Adjusted clinical burden, mean (95% CI)			
Variables	COPD-only (*n* = 366)	COPD-anxiety and/or depression (*n* = 213)	*p* value	COPD-only (*n* = 184)	COPD-anxiety and/or depression (*n* = 184)	IRR	*p* value
Annual number of COPD-related outpatient visits	1.00 (0.33, 2.33)	1.33 (0.66, 2.66)	0.028	1.59 (1.33, 1.86)	2.54 (2.02, 3.07)	0.47 (0.27, 0.66)	<0.001
Annual number of COPD-related hospitalization	0.33 (0.33, 0.66)	0.66 (0.33, 1.00)	<0.001	0.59 (0.52, 0.65)	0.78 (0.69, 0.87)	0.29 (0.16, 0.42)	<0.001
Length of COPD-related hospitalization	4.00 (3.00, 7.00)	6.00 (3.00, 11.00)	<0.001	5.61 (4.86, 6.34)	7.74 (6.72, 8.76)	0.32 (0.16, 0.47)	<0.001

IQR, interquartile range; IRR, incidence rate ratio; CI, confidence interval.

When clinical burden was adjusted for demographic and clinical characteristics, the annual number of COPD-related outpatient visits was 59.74% (incidence rate ratio [IRR] =0.47; 95% confidence interval [CI]: 0.27, 0.66) higher in the COPD-anxiety and/or depression group compared with the COPD-only group. At the same time, the annual number of COPD-related hospitalizations and the annual length of COPD-related hospitalizations were 32.20% (IRR = 0.29; 95% CI: 0.16–0.42) and 37.97% (IRR = 0.32; 95% CI: 0.16–0.48) higher, respectively, in the COPD-anxiety and/or depression group (Table 2).

### 4.3 Economic burden of anxiety and/or depression among COPD patients

The median annual total healthcare cost in the COPD-anxiety and/or depression group was CNY 7224.49 (quartile 2 to quartile 3: CNY 3771.36–4079.84), while in the COPD-only group it was CNY 5218.59 (quartile 2 to quartile 3: CNY 3097.75–9934.68), indicating that the annual COPD-related total healthcare costs were significantly higher in COPD-anxiety and/or depression group. Similarly, the COPD-related medical and pharmacy costs were also higher for the COPD-anxiety and/or depression group versus the COPD-only group, with the differences in the median value of annual COPD-related medical costs (CNY 4429.19 vs. CNY 3148.14) and the median value of annual COPD-related pharmacy costs (CNY 2467.45 vs. CNY 2014.53) (Table 3).

**Table 3:** Unadjusted and adjusted economic burden of anxiety and depression among patients with COPD.

	Unadjusted economic burden, median (IQR)			Adjusted economic burden, mean (95% CI)			
Variables	COPD-only (*n* = 366)	COPD-anxiety and/or COPD-depression (*n* = 213)	*p* value	COPD-only (*n* = 184)	COPD-anxiety and/or COPD-depression (*n* = 184)	IRR	*p* value
Annual COPD-related medical costs, (CNY)	3148.14 (1848.09, 6097.37)	4429.19 (2169.46, 8871.61)	<0.001	4792.69 (4030.79, 5554.59)	6627.60 (5662.93, 7592.28)	0.32 (0.15, 0.49)	<0.001
Annual COPD-related pharmacy costs, (CNY)	2014.53 (1010.96, 3761.70)	2467.45 (1194.42, 5098.35)	0.001	2936.22 (2500.54, 3371.90)	3972.28 (3345.81, 4598.75)	0.30 (0.12, 0.48)	0.001
Annual total healthcare costs, (CNY)	5218.59 (3097.75, 9934.68)	7224.49 (3771.36, 14079.84)	<0.001	7729.09 (6575.43, 8882.75)	10599.89 (9068.53, 12131.24)	0.32 (0.15, 0.48)	<0.001

IQR, interquartile range; CNY, Chinese Yuan; IRR, incidence rate ratio; CI, confidence interval.

After adjustment for all other factors that may influence healthcare expenditures, the COPD-anxiety and/or depression group was still associated with higher healthcare costs compared with the COPD-only group. The adjusted mean annual total healthcare cost in the COPD-anxiety and/or depression group was CNY 10599.89 (95% CI: 9068.53–12131.24), while in the COPD-only group, the mean cost was CNY 7729.09 (95% CI: 6575.43–8882.75). The total healthcare costs were 37.13% (IRR = 0.32; 95% CI: 0.15–0.48) higher in the COPD-anxiety and/or depression group. Furthermore, this group showed higher mean annual COPD-related medical costs (CNY 6627.60; 95% CI: 5662.93–7592.28) and mean annual COPD-related pharmacy costs (CNY 3972.28; 95% CI: 3345.81–4598.75) (Table 3).

## 5 Discussion

The COPD-AD China Registry is a nationwide registration study with a comprehensive database in China to track COPD patients with anxious and depressive symptoms. Its goal is to screen for anxiety and depression among COPD patients at an early stage, and to track how these conditions affect the overall progression and prognosis of COPD. In this study, we further analyzed this database and found that older adult COPD patients with anxious and depressive symptoms had an increased clinical burden, including higher COPD-related outpatient visits, more COPD-related hospitalizations, and longer COPD-related hospitalizations. Moreover, they also had higher healthcare costs, including medical and pharmacy costs.

Anxiety and depression are common comorbidities among older adult COPD patients. Our previous study showed that these significantly reduce quality of life and are associated with a greater burden of symptoms and exacerbations among COPD patients (4). However, studies on the clinical burden of COPD-related outpatient visits, COPD-related hospitalizations, and length of COPD-related hospitalizations caused by anxiety and depression remain very limited in China. Dalal et al. estimated the clinical burden of depression/anxiety in COPD patients using data from a comprehensive source of medical and pharmacy claims from the IMS LifeLink Database in the United States, and found that COPD patients with depression/anxiety have a significantly higher risk of acute exacerbations (23). Our study further confirms that anxiety and depression not only increase the risk of COPD exacerbations, but also increase the number of outpatient visits and extend the length of hospital stay, thereby increasing medical resource utilization in older adult COPD patients. Studies have shown that anxiety and depression can activate the sympathetic nervous system and the hypothalamic–pituitary–adrenal axis in COPD patients, causing a decrease in immunity, thus increasing the risk of respiratory infection and exacerbations (32, 33). Additionally, they are associated with disinterest in self-care, poorer adherence to treatment, and a higher probability of continuing to smoke, contributing to poor control and deterioration of COPD (34, 35). Moreover, they can result in cognitive function impairment, making patients more sensitive to or likely to report respiratory symptoms. This may lead to more frequent doctor visits and increased opportunities to be prescribed medication to manage their symptoms (13, 36). These findings suggest that anxiety and/or depression can increase the risk of outpatient visits, hospitalizations due to acute exacerbation of COPD, and length of hospitalizations in older adult COPD patients. Hence, we believe that anxiety and/or depression increase the clinical burden among older adult COPD patients.

In this study, we also examined the healthcare costs of older adult COPD patients caused by anxiety and depression. We found that co-anxiety or co-depression contributed to increased pharmacy costs among older adult COPD patients. COPD is a chronic disease that requires long-term treatment and management, including drug therapy, oxygen therapy, rehabilitation training, and other therapeutic means (37). The cost of these treatments can place a significant financial burden on patients and families. When COPD patients suffer from anxiety and/or depression, they can complicate the course of COPD. A causal relationship has been reported between anxiety or depression in COPD patients and changes in routine use of pharmacies, including bronchodilators and inhaled steroids (38). A Spanish cohort study also showed that the presence of anxious and depressive symptoms increased the use of antibiotics and systemic corticosteroids in COPD patients (39). These studies suggest that co-psychiatric symptoms are associated with increased pharmacy use in COPD patients. It may be that anxiety and depression can cause worsening symptoms and frequent acute exacerbations, resulting in increased use of pharmacies. In addition, our study also found that older adult COPD patients co-morbid with anxiety or depression had higher medical costs, including examination fees, nursing fees, and surgery fees.

On one hand, anxiety or depression can directly affect the health status of COPD patients. Symptoms of anxiety or depression in COPD patients are directly related to continued smoking, increased burden of symptoms, poorer physical and social functioning, and difficulty with daily activities (21, 40). Depressed states also damage the immune system, making them vulnerable to infections that increase the frequency of exacerbations (41). Hence, COPD patients with psychiatric symptoms had more severe clinical symptoms, poorer quality of life, and high risk of acute exacerbation, thus increasing the risk of complications that require more medical resources and time to treat (39, 42–44).

On the other hand, anxious and depressive symptoms can also negatively affect the mental state and behavior of COPD patients, leading to more unnecessary medical procedures (21, 45, 46). In general, patients with anxiety or depression rated their health as worse than that of the general population, including its impact on quality of life and functional status (47). This low confidence in their own health and the effectiveness of treatment can lead to a reduced ability to cope with chronic diseases (48). As a result, anxiety and/or depression may affect a patient’s ability to provide informed consent and understand whether to accept or reject a particular treatment, thus affecting their treatment compliance (49, 50). In addition, anxious and depressive symptoms have also been associated with an increased perception of breathing difficulties (51). This may result in unnecessary hospital visits and increased use of bronchodilators, inhaled and systemic corticosteroids, and antibiotics (49). All of these may result in more examinations or nursing interventions.

Our study provides a new direction on how to control healthcare costs for older adult COPD patients by focusing on the mental health of COPD patients and early intervention and treatment of psychiatric symptoms, which may be beneficial in reducing healthcare costs. Therefore, psychiatric symptoms should be included in routine screening in the health management of older adult COPD patients to provide appropriate psychiatric treatment or prompt referral to mental health services for this population.

Nonetheless, there are limitations to our study. First, as with any observational study, our results may not be applicable to older adult COPD patients in the general population or in other clinical environments. This study included only older adult COPD patients admitted to the Respiratory Department of the Affiliated Hospital of Guangdong Medical University. Second, even though matching and multivariate adjustment were conducted, the possibility remains that the groups were not fully matched on all unobserved characteristics (i.e., residual confounding). Third, other comorbidities and severe symptoms of COPD may increase the risk of anxiety and depression, and their potential impact on the economic burden of COPD patients cannot be excluded. Finally, the healthcare costs may have been underestimated, as our study did not delve into indirect economic burdens, such as the loss of working time of caregivers and the heavy mental burdens caused by illness and disability to the patients and their families.

In summary, our study found that anxious and/or depressive symptoms were significantly associated with more severe clinical and economic burdens in older adult COPD patients. In the management of chronic diseases such as COPD, routine screening of psychiatric symptoms should be an integral part of clinical care to diagnose and treat anxious and depressive symptoms in a timely manner, and to provide mental health counseling services and prompt referrals for mental health services. Further understanding of the impact of comorbidities such as anxiety and depression on the assessment and management of COPD may be beneficial for controlling the healthcare costs of chronic diseases and guiding the formulation of health insurance policies.

## Data availability statement

The raw data supporting the conclusions of this article will be made available by the authors, without undue reservation.

## Ethics statement

The studies involving human participants were approved by Ethics Committee of Affiliated Hospital of Guangdong Medical University. The studies were conducted in accordance with the local legislation and institutional requirements. The patients/participants provided their written informed consent to participate in this study.

## Author contributions

DW: supervision. XZ: data curation, writing-original draft preparation. GL: writing-original draft preparation. DL: formal analysis, methodology. LZ: investigation. QH: data curation. MC: writing-reviewing and editing. DL: conceptualization and methodology. BW: conceptualization and methodology. DH: formal analysis and validation. HW: visualization, investigation, data curation. All authors agreed to be accountable for the content of the work, and approved the final manuscript.

## Footnotes

^1^mzf.fwncpc.com
